# Enhancing nursing students’ patient-centeredness attitudes and emotional skills through co-teaching with patients and caregivers: A mixed-methods study

**DOI:** 10.1371/journal.pone.0332510

**Published:** 2025-09-29

**Authors:** Sara Alberti, Matías Eduardo Díaz Crescitelli, Loris Bonetti, Sergio Rovesti, Paola Ferri

**Affiliations:** 1 Department of Biomedical, Metabolic and Neural Sciences, University of Modena and Reggio Emilia, Modena, Italy; 2 Laboratorio EduCare, University of Modena and Reggio Emilia, Modena, Italy; 3 Nursing Research Competence Centre, Ente Ospedaliero Cantonale, Bellinzona, Switzerland; 4 Department of Business Economics, Health and Social Care, University of Applied Sciences and Arts of Southern Switzerland, Manno, Switzerland; University of Verona, ITALY

## Abstract

The objective of this study is to evaluate changes in patient-centeredness attitudes, empathy, and emotional intelligence among nursing students trained with patients and caregivers as co-teachers. A mixed-methods embedded study was conducted. The quantitative strand consists of a quasi-experimental design, measuring third-year nursing students’ scores before and after an educational intervention, compared with first- and second-year students’ scores. Data were collected using three validated scales: the Patient-Practitioner Orientation Scale (PPOS-8-IT), Jefferson Scale of Empathy Health Professions Student (JSEHPS), and Self-Report Emotional Intelligence Test (SREIT). The qualitative strand explored student perceptions through focus group post-intervention. Quantitative and qualitative data were initially analyzed separately, then triangulated during interpretation. Results showed statistically significant improvements in scale scores post-intervention (n = 146). Three themes emerged from the qualitative analysis, aligning with quantitative findings. Patient-centeredness attitudes among students increased significantly after the intervention (PPOS-8-IT mean difference = 0.26, p < 0.001). Mixed-methods analyses highlight the importance of a participatory approach to decision-making and sharing clinical information. Empathy (JSEHPS mean difference = 6.39, p < 0.001) and emotional intelligent (SREIT mean difference = 3.19, p < 0.001) also increased post-intervention. Understanding patients’ emotional states and managing one’s own emotions are integral to patient-centered practice, emphasizing the need for healthcare professionals to balance empathy with professional boundaries. Interestingly, our study found that outcomes increase over the three years of the course (n = 424). The analyses reveal that the difference among the three years is statistically significant both for the patient-centeredness attitudes (PPOS-8-IT H(2)=55.99, p < 0.001) and empathy (JSEHPS H(2)=37.78, p < 0.001).

## Introduction

Patient-Centered Care (PCC) is a bio-psychosocial care model that considers the person as a whole, acknowledging their health, emotional, social, spiritual, occupational, and physical needs [1].

This model aligns with the broader movement to humanize healthcare by actively involving patients and families in care processes [2]. PCC emerged as a response to the limitations of the traditional biomedical model, which often overlooks the individual’s subjective experience. Both models shape medical practice, especially the nature of the healthcare professional–patient relationship.

A central concept in PCC is patient-centeredness, which has been recently defined as “an approach to care that is respectful of and responsive to the preferences, needs, and values of patients, ensuring that patient values guide all clinical decisions” [3]. Patient-centeredness includes five core dimensions: a bio-psychosocial perspective, recognition of the patient as a person, sharing, the formation of a therapeutic alliance, and the recognition of the healthcare professional as a person [1,3]. Among the dimensions used to measure patient-centeredness in research, sharing and caring play a central role. Sharing refers to the willingness to adopt a participatory approach to care, including shared decision-making and the exchange of clinical information. Caring refers to the healthcare professional’s openness to the patient’s expectations, emotions, and life circumstances as essential elements of the therapeutic process, reflecting a bio-psychosocial perspective [4,5]. More recent frameworks extend this concept by including empathy among its essential principles [6].

Empathy, a core competence for healthcare professionals, has been recently defined as “the ability to understand and share another person’s perspectives and feelings, and to communicate this understanding with the intent to help” [7]. It involves both cognitive and affective components. The cognitive domain refers to the capacity to intellectually comprehend the patient’s perspective, while the affective domain involves the ability to emotionally resonate with the patient’s feelings [7,8]. Empathy supports the delivery of emotional support, enhances communication, and fosters trust within the therapeutic relationship [3].

Empathy and patient-centeredness have been linked to numerous positive outcomes, including higher care quality, improved professional–patient relationships, greater patient engagement, better adherence to treatment, increased patient satisfaction, and overall quality of life [8–14]. Furthermore, empathy is associated with lower emotional distress among students and healthcare professionals [15–17]. Empirical studies confirm that empathy and patient-centeredness are significantly correlated, with empathy often considered a precursor of patient-centered attitudes [18, 19].

Despite their importance, both empathy and patient-centeredness show a decline during healthcare training, including in nursing education [20–24]. This decrease is partly attributed to the persistence of the biomedical model and the lack of specific training promoting these competencies [12,25]. Other influencing factors include age, gender, and emotional intelligence [26].

Emotional intelligence, distinct from empathy, is defined as “the capacity to reason about emotions, to perceive, understand, and manage emotions in oneself and others, and to use this knowledge to guide thinking and behavior” [27]. Emotional intelligence plays a key role in handling the emotional demands of healthcare work [28,29], and is positively associated with empathy [26]. In nursing, research shows that emotional intelligence levels can evolve throughout the training period and highlights the importance of incorporating its development into curricula [30,31]. While associations between empathy, emotional intelligence, and patient-centeredness are well-documented, their interrelationships and development trajectories over time remain insufficiently explored.

Importantly, these competencies—patient-centeredness, empathy, and emotional intelligence—can be taught and enhanced [32]. Educational interventions that involve patients as co-teachers have been shown to foster patient-centeredness and empathy [6]. However, recent theoretical frameworks suggest that while co-teaching with patients and caregivers enhances the educational experience, further research is needed to understand the mechanisms through which patient involvement promotes learning [33]. In particular, although co-teaching appears to support empathy and patient-centeredness, its impact on emotional intelligence remains unclear [23]. Moreover, it is not yet known whether and how these competencies evolve over time when such teaching methods are introduced. This study aims to fill this gap.

### Study Objective

Based on the hypothesis that involving the patients and caregivers as co-teachers promotes patient-centeredness and the development of emotional skills, the objective of the present study is to evaluate the impact of an educational intervention co-led by patients, caregivers, and nurse educators on nursing students’ patient-centeredness attitudes, empathy, and emotional intelligence.

To address this objective, the study was designed to answer the following research questions, aligned with the methods adopted:

- Primary quantitative question: What is the impact of education with patients and caregivers as teachers on patient-centeredness attitudes, empathy, and emotional intelligence in nursing students?
- Secondary qualitative question: How do students perceive changes in their patient-centeredness attitudes, empathy, and emotional intelligence following the intervention?
- Tertiary quantitative question: Compared to students from other academic years not exposed to the intervention, how do patient-centeredness attitudes, empathy, and emotional intelligence differ?

## Materials and methods

### Study design

To ensure alignment between objectives and methods, a mixed-methods embedded design was adopted. This approach integrates a qualitative component within a quasi-experimental quantitative design, allowing for both the measurement of predefined outcomes and the exploration of participants’ subjective experiences [34,35].

The quantitative strand addresses the primary and tertiary research questions by assessing changes in patient-centeredness attitudes, empathy, and emotional intelligence in third-year nursing students before and after the intervention. Additionally, it compares these outcomes with those of first- and second-year students who did not receive the intervention. This comparison allows for contextualizing the intervention’s impact within the broader educational trajectory and exploring the potential for curriculum-wide implementation.

The qualitative strand addresses the secondary research question by exploring students’ perceptions and experiences related to the development of emotional competencies and patient-centeredness after the intervention. Data were collected through focus groups conducted with participating students, providing insights that complement and enrich the quantitative findings.

**Fig 1:**
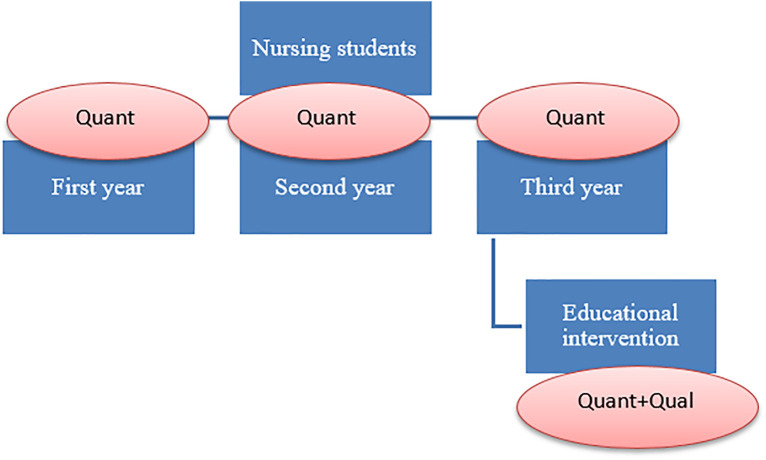
Study Design.

The rationale for employing a mixed-methods design lies in the need to obtain a comprehensive and nuanced understanding of the effects of the intervention, combining measurable outcomes with students’ subjective experiences [36]. This approach enhances completeness and credibility, consistent with Bryman’s classification of mixed-methods purposes [37]. Fig 1 depicts the study design.

### Sampling and eligibility criteria

The study was conducted on students enrolled in the Nursing Degree Program of Modena at the University of Modena and Reggio Emilia.

Inclusion Criteria:

- Students enrolled in the first, second, or third year of the Nursing Degree Program in the Academic Year 2023–2024
- Students attending the course at the Modena campus.

A Power Analysis using GPower 3.1 software resulted in a minimum sample size of 128 participants required to obtain medium effects with sufficient power (1-β = 0.80) and a significance level of 0.05. The recruitment started on October 2, 2023, and ended on October 31, 2023. On October 2, third-year students were recruited. After being informed about the study and providing informed consent, those who agreed to participate were administered the scales as a pre-intervention assessment. The post-intervention data collection for third-year students was conducted immediately after the completion of the educational sessions, which took place on October 5 and 6, 2023. First- and second-year students, who did not participate in the educational intervention, were recruited in a subsequent phase—on October 20 and October 31, respectively.

### Educational intervention and level of patient and caregiver involvement

In October 2023, third-year students were divided into 2 groups, and each participated in a 4-hour workshop titled ‘ComuniCare: partnership between the cared-for and caregiver.’ The workshop syllabus is detailed in S1 File. The course aimed to learn patient-centeredness attitudes and emotional competencies and was conducted by 5 nurse educators, a patient diagnosed with multiple sclerosis, and an informal caregiver of a woman who had suffered hemorrhagic stroke sequelae specifically trained to co-conduct educational interventions for healthcare professionals through an advanced course. The patient and caregiver engagement, in this case, corresponds to the fourth degree of the Spectrum of Involvement: ‘Patients and Caregivers-teachers are involved in teaching’. The workshop was co-designed with both the patient and the informal caregiver, taking into account their health conditions and capabilities. We ensured the classroom was free of physical barriers, and while remote participation via Teams was arranged as a backup, both the patient and the caregiver were able to attend the workshop in person. Special attention was also given to the caregiver’s needs, including scheduling the workshop at a time compatible with their caregiving responsibilities. The workshop was structured mainly in two phases: a first phase of storytelling in which the patient and caregiver shared their story with the students in the form of a narrative interview co-conducted with a tutor, and a second phase of group work, in which students, based on the stories heard, responded to some open-ended questions about the relationship and communication between patient and healthcare professional.

### Methods and data collection tools

#### Methods and tools for quantitative data collection.

Quantitative data were collected from recruited students using the following paper-based questionnaires: The Patient-Practitioner Orientation Scale (PPOS-8-IT), Jefferson Scale of Empathy Health Profession Student (JSEHPS), Self-Report Emotional Intelligence Test (SREIST), and a participant socio-demographic form. Data collection took place at the beginning of the academic year in October 2023: third-year students completed the questionnaires both before and after the educational intervention. First and second-year students completed the questionnaires only once afterward.

Specifically, the data collection tools used were:

- The Patient-Practitioner Orientation Scale (PPOS-8-IT): This is a self-report scale translated and validated in Italian [5]. It consists of 8 items divided into two dimensions, ‘Sharing’ (items 1,3,5,7) and ‘Caring’ (items 2,4,6,8). Items are rated on a Likert-type scale ranging from 1 (Totally Disagree) to 6 (Totally Agree). The Cronbach’s α reported in the literature is 0.890.
- Jefferson Scale of Empathy Health Profession Student (JSEHPS): This is an empathy measurement scale translated into Italian and validated on nursing students [38]. It consists of 20 items, and participants rate their level of agreement on a Likert scale ranging from 1 (Strongly Disagree) to 7 (Strongly Agree). The total score ranges from a minimum of 20 to a maximum of 140: higher scores indicate higher levels of empathy. The Cronbach’s α reported in the literature varies between 0.80–0.89, and in the Italian version α = 0.78.
- Self-Report Emotional Intelligence Test (SREIST): This is a self-report measurement tool of emotional intelligence translated and validated in Italian. It consists of 33 items rated on a 5-point Likert scale, with 1 representing total disagreement and 5 representing complete agreement [39]. The total score ranges from a minimum of 33 to a maximum of 165: higher scores indicate higher levels of emotional intelligence. The Cronbach’s α of the scale validated in Italian is 0.89.
- Participant Socio-demographic Form: The questionnaire contained questions regarding age, gender and nationality useful for describing the sample.

#### Methods and tools for qualitative data collection.

Among the third-year students who participated in the workshop, many expressed their willingness to take part in a Focus Group. However, due to limited resources, it was only possible to conduct a single session. Therefore, 12 students were randomly selected from the pool of volunteers, following the principle of equality, to ensure that all interested participants had an equal opportunity to be included. Although random sampling is not typically used in qualitative research, in this case it was adopted as a pragmatic and impartial solution to manage high participation interest within logistical constraints [40].

The Focus Group aimed to investigate students’ perceptions and attitudes regarding patient-centeredness in the care process, the role of emotions, and education with the patient-as-teacher. It was conducted by a researcher experienced in qualitative research (MC) using a guide with semi-structured open-ended questions, developed based on the objectives of our study and consultation of various studies in the literature (S2 Appendix). The entire group interview was audio-recorded, to allow for transcription and data analysis.

### Data analysis

#### Quantitative data analysis.

The data were analyzed using SPSS version 29. Descriptive statistics, such as frequencies, percentages, means, and standard deviations (SD), were employed to summarize participant characteristics and scores obtained from the demographic form and administered scales. The normality of the distribution of scores obtained from the three scales was then assessed using Shapiro-Wilk and Kolmogorov tests to determine the most appropriate statistical techniques (parametric or non-parametric) for subgroup comparisons. Statistical significance was defined as p < 0.05.

#### Qualitative data analysis.

The focus group was audio-recorded with participants’ consent. Thematic analysis, as described by Braun and Clarke (2016) [41], was conducted by three researchers (SA, MC, PF). The main steps are outlined below:

1. Transcription of verbatim recordings and full reading (SA).
2. Subdivision into conversation sequences and definition of initial labels (SA).
3. Combination of labels to identify main themes and sub-themes (SA and MC).
4. Review of the identified issue list to ensure internal consistency (SA, MC, PF).
5. Description of main themes and writing of results reports (SA, MC, PF).

#### Integration of data.

Initially, quantitative and qualitative data were analyzed separately and independently. Subsequently, during the interpretation phase, the findings from both methods were triangulated. Quantitative and qualitative findings were juxtaposed, and convergences, discrepancies, and complementarities were identified [42]. Bringing together all information allowed for data integration using Joint Display [34,43]. Fetters et al. (2013), defined Joint Displays as a way to ‘integrate the data by bringing the data together through a visual means to draw out new insights beyond the information gained from the separate quantitative and qualitative results’ [43]. Following some examples reported in the literature [44,45] we created a table in which we linked qualitative themes with the quantitative results. Then we added a column with a comment and integrated the findings in a descriptive way.

### Ethical considerations

The present study was approved by the local ethics committee of Area Vasta Emilia Nord (PROT. AOU 0022028/23 of July 19, 2023) and conducted following the principles of the World Medical Association Declaration of Helsinki (1964). All students were informed that their participation in the study was voluntary and they were free to withdraw at any time without affecting their academic course. Written consent of all participants was obtained. All students were further assured that their information would be kept confidential. The researchers took care to ensure that students did not feel pressured while responding.

## Results

### Quantitative analysis pre- and post-educational intervention (first research question)

#### Description of the sample.

A total of 146 third-year nursing students were recruited, with a study participation rate of 98% (number of eligible students = 149). The majority of students who participated in the educational intervention were females (79.45%) and of Italian nationality (91.78%). The average age of the students was 24.03 years ± 6.06, ranging from 20 to 59 years.

#### Results of the PPOS-8-IT scale.

We tested the effectiveness of the educational intervention in terms of developing patient-centered attitudes by comparing the mean values of the PPOS-8-IT scale administered before and after the intervention.

Assuming the normal distribution of data from the scale (Shapiro-Wilks test p = 0.139; Kolmogorov test p = 0.200), we used a paired t-test to compare the means of pre- and post-intervention scores.

Table 1 presents the results of the scale for individual items, subscales, and total scale.

**Table 1:** Results of the PPOS-8-IT scale pre- and post-intervention.

PPOS-8-IT: items and subscales	PRE-		POST-		MD	t (df)	p-value
	m	SD	m	SD			
Item 1	4.34	1.10	4.67	1.12	0.33	−3.19 (136)	0.002*
Item 2	3.72	1.36	4.09	1.38	0.37	−3.58 (136)	<0.001*
Item 3	3.03	1.48	3.33	1.54	0.30	−2.38 (135)	0.019*
Item 4	5.49	0.96	5.49	1.09	0.007	−0.06 (137)	0.949
Item 5	5.16	1.27	5.43	1.13	0.27	−2.42 (137)	0.017*
Item 6	4.91	1.21	5.08	1.23	0.17	−1.43 (137)	0.155
Item 7	2.95	1.58	3.28	1.57	0.33	−2.50 (137)	0.014*
Item 8	5.46	0.85	5.64	0.68	0.18	−2.54 (137)	0.012*
Subscale ‘Sharing’	3.86	0.82	4.17	0.88	0.31	−4.45 (135)	<0.001*
Subscale ‘Caring’	4.90	0.66	5.08	0.65	0.18	−3.35 (136)	0.001*
PPOS-8-IT Total score	4.38	0.60	4.64	0.62	0.26	−5.35 (134)	<0.001*

Legend: m = mean; SD = Standard Deviation; MD = mean difference; t = Student’s t-test; dl = degrees of freedom; *p < 0.05.

The results show a statistically significant difference between the pre- and post-intervention total scores on the PPOS-8-IT scale (MD = 0.26, p < 0.001), as well as the scores on the individual subscales ‘Caring’ (MD = 0.18, p = 0.001) and ‘Sharing’ (MD = 0.31, p < 0.001), with an increase in scores favoring the intervention; furthermore, the ‘Caring’ subscale exhibits higher scores compared to the ‘Sharing’ subscale. Analyzing the individual items, the mean scores are statistically higher in the post-intervention except for item 4, where the difference is almost negligible (MD = 0.007, p = 0.949), and item 6, where the post-intervention scores are higher than the pre-intervention scores but not statistically significant (mean difference = 0.17, p = 0.155).

#### Results of the JSEHPS scale.

Reverse scoring was first performed for items 1, 3, 6, 7, 8, 11, 12, 14, 18, as indicated in the Jefferson Scale of Empathy Scoring Algorithm, before proceeding with the statistical analysis of the data [26].

Due to the non-normal distribution of the data (Shapiro-Wilks test p < 0.001; Kolmogorov test p < 0.001), the non-parametric Wilcoxon signed-rank test was used for the pre- and post-intervention comparison.

Table 2 shows the results of the statistical tests for individual items, subscales, and the total scale.

**Table 2:** Results of the JSEHPS scale pre- and post-intervention.

JSEHPS scale: items and total score	PRE-				POST-				MD	z	p-value
	M	IQR	m	SD	M	IQR	m	SD			
Item 1	6.00	2.00	5.98	1.27	7.00	0.00	6.50	1.21	0.52	−4.60	<0.001*
Item 2	7.00	1.00	6.38	0.94	7.00	1.00	6.49	0.94	0.11	−1.03	0.303
Item 3	4.00	2.00	3.87	1.22	4.00	1.00	4.42	1.22	0.55	−4.26	<0.001*
Item 4	7.00	1.00	6.25	1.11	7.00	1.00	6.40	1.04	0.15	−1.60	0.110
Item 5	5.00	2.00	4.68	1.44	6.00	2.00	5.86	1.15	1.18	−7.69	<0.001*
Item 6	4.00	2.00	3.85	1.39	4.00	2.00	4.21	1.52	0.36	−2.45	0.014*
Item 7	7.00	0.00	6.64	0.81	7.00	0.00	6.65	0.98	0.01	−0.21	0.836
Item 8	6.00	1.00	6.01	1.07	7.00	1.00	6.37	0.96	0.36	−3.27	0.001*
Item 9	6.00	2.00	6.12	1.09	7.00	1.00	6.22	1.09	0.10	−1.83	0.068
Item 10	6.00	1.00	6.14	1.10	7.00	1.00	6.48	0.81	0.34	−3.85	<0.001*
Item 11	6.50	1.00	6.21	1.00	7.00	1.00	6.29	1.10	0.08	−10.10	<0.001*
Item 12	6.00	2.00	5.98	1.07	7.00	1.00	6.08	1.36	0.10	−9.89	<0.001*
Item 13	7.00	1.00	6.30	1.01	7.00	1.00	6.51	0.88	0.21	−2.22	0.026
Item 14	7.00	1.00	6.35	1.11	7.00	0.00	6.58	0.98	0.23	−10.26	<0.001*
Item 15	6.00	1.00	5.86	1.38	6.00	1.00	5.90	1.52	0.04	−1.02	0.309
Item 16	6.00	1.00	6.14	1.08	7.00	1.00	6.41	0.95	0.27	−3.17	0.002*
Item 17	5.00	2.00	4.71	1.46	6.00	2.75	5.38	1.38	0.67	−4.66	<0.001*
Item 18	3.00	2.00	2.97	1.40	4.00	2.00	3.71	1.57	0.74	−4.23	<0.001*
Item 19	6.00	3.00	5.65	1.63	6.00	2.00	5.83	1.47	0.18	−1.65	0.099
Item 20	7.00	1.00	6.16	1.12	7.00	1.00	6.44	0.96	0.28	−3.40	0.001*
JESHPS Total score	114.00	16.00	111.94	11.74	120.50	13.75	118.34	12.58	6.39	−7.62	<0.001*

Legend: M = median; IQR = Interquartile Range; m = mean; SD = Standard Deviation; MD = mean difference; z = Wilcoxon test; *p < 0.05.

The results show a statistically significant difference (MD = 6.39, p < 0.001) between the pre- and post-intervention total scores of the JSEHPS scale, with an increase in scores favoring the intervention. Analyzing individual items, the scores are statistically higher in the post-intervention except for items 2, 4, 7, 9, 15, and 19, where the scores in the post-intervention are higher than the pre-intervention but not statistically significant.

#### Results of the SREIT scale.

Assuming normality of data distribution for the scale (Shapiro-Wilks test p = 0.458; Kolmogorov test p = 0.200), we used a paired sample t-test to compare the means of pre- and post-intervention scores.

Table 3 shows the results of the statistical analyses of the scale with the pre- and post-intervention comparison.

**Table 3:** Results of SREIT scale pre- and post-intervention.

SREIT: items e total score	PRE-		POST-		MD	t(df)	p-value
	m	SD	m	SD			
Item 1	3.82	0.92	4.05	0.76	0.23	−2.77 (136)	0.006*
Item 2	3.99	0.82	4.02	0.77	0.03	−0.561 (137)	0.576
Item 3	3.41	0.91	3.58	0.93	0.17	−2.47 (137)	0.015*
Item 4	4.04	0.72	4.09	0.77	0.05	−1.02(135)	0.309
Item 5	2.08	0.94	2.05	0.98	−0.03	0.34 (133)	0.733
Item 6	4.40	0.74	4.43	0.65	0.03	−0.33 (135)	0.740
Item 7	3.51	0.93	3.43	0.94	−0.08	0.92 (137)	0.359
Item 8	4.20	0.95	4.34	0.78	0,14	−1.95 (135)	0.053
Item 9	3.92	0.94	3.97	0.92	0.05	−0.63 (136)	0.530
Item 10	3.46	1.09	3.46	1.00	0.007	0.12 (137)	0.905
Item 11	3.25	1.17	3.41	1.11	0.16	−2.44 (137)	0.016*
Item 12	3.16	0.99	3.20	0.90	0.04	−0.57 (136)	0.571
Item 13	3.36	1.09	3.50	1.10	0.14	−1.91 (137)	0.059
Item 14	4.12	0.76	4.30	0.65	0.18	−2.97 (137)	0.004*
Item 15	3.75	1.01	3.88	0.95	0.13	−1.59 (137)	0.115
Item 16	3.69	0.80	3.85	0.84	0.16	−2.89 (136)	0.005*
Item 17	3.99	0.81	4.08	0.80	0.09	−1.30 (136)	0.197
Item 18	4.10	0.82	4.20	0.76	0.10	−1.71 (137)	0.090
Item 19	3.53	1.08	3.56	0.98	0.03	−0.35 (137)	0.725
Item 20	3.95	0.80	3.83	0.88	−0.12	1.80 (136)	0.074
Item 21	3.04	1.13	3.16	1.03	0.12	−1.65 (136)	0.100
Item 22	3.63	0.95	3.79	0.89	0.16	−2.69 (134)	0.008*
Item 23	3.80	0.84	3.77	0.88	−0.03	0.33 (136)	0.740
Item 24	4.23	0.83	4.41	0.69	0.18	−2.71 (137)	0.008*
Item 25	3.94	0.93	4.07	0.88	0.13	−1.58 (137)	0.116
Item 26	3.52	1.03	3.78	0.90	0.26	−3.69 (137)	<0.001*
Item 27	3.18	0.94	3.46	0.87	0.28	−3.84 (135)	<0.001
Item 28	2.12	0.99	2.20	1.03	0.08	−0.88 (137)	0.379
Item 29	3.63	0.82	3.74	0.83	0.11	−1.82 (135)	0.071
Item 30	4.01	0.73	4.07	0.71	0.06	−1.00 (137)	0.319
Item 31	3.27	1.08	3.60	0.96	0.33	−4.61 (137)	<0.001*
Item 32	3.88	0.78	4.04	0.74	0.16	−2.61 (136)	0.010*
Item 33	2.46	1.09	2.40	1.04	−0.06	0.58 (137)	0.561
SREIT Total score	111.51	14.12	114.70	14.03	3.19	−5.10 (137)	<0.001*

Legend: m = mean; SD = Standard Deviation; MD = mean difference; t = Student’s t-test; dl = degrees of freedom; *p < 0.05.

Results show a statistically significant difference between pre- and post-intervention with an increase in the total score in favor of the intervention, indicating a higher level of emotional intelligence (MD = 3.19, p < 0.001). However, when analyzing individual items, 12 items show a statistically significant increase in score in the post-intervention (p < 0.05), while 19 items show a non-statistically significant increase (p > 0.05); the scores of items 1, 2, and 33 are lower in the post-intervention but not statistically significant.

An ANCOVA between-subjects’ analysis was conducted to assess the interaction effect between the variables. Specifically, the analysis aimed to examine the effect of gender, nationality, and workshop date on the post-intervention scores, while controlling for the pre-intervention scores, and to determine whether there were meaningful differences between subgroups of these variables.

The results show that the differences in mean scores on the PPOS-8-IT, JESHPS, and SREIT scales were not statistically significant with respect to gender (PPOS-8-IT: F(1) = 1.07, p = 0.302; JESHPS: F(1) = 0.26, p = 0.613; SREIT: F(1) =0.20, p = 0.659), nationality (PPOS-8-IT: F(1) = 3.89, p = 0.05; JESHPS: F(1) = 0.11, p = 0.736; SREIT: F(1) =0.03, p = 0.855), or workshop date (PPOS-8-IT: F(1) = 0.91, p = 0.341; JESHPS: F(1) =0.06, p = 0.804; SREIT: F(1) =1.81, p = 0.180). Therefore, the interaction effect between the intervention and these variables was not significant.

Additionally, a simple linear regression was conducted to assess whether age predicts scale scores. The model was not statistically significant for any of the scales (PPOS-8-IT: F(1) = 0.289, p = 0.592; JESHPS: F(1) = 1.044, p = 0.309; SREIT: F(1) = 0.455, p = 0.501).

These findings suggest that the observed effects are consistent across the demographic and background characteristics assessed, and are not confounded by these variables.

### Qualitative data analysis (second research question)

From the qualitative analysis, three themes have been identified, as follows: ‘Attitudes favoring patient-centeredness’, ‘The emotional bond in the care relationship’, ‘Learning from the patient’s history.’

#### Attitudes favoring patient-centeredness.

Table 4 presents the sub-themes, labels and quotes related to the theme ‘Attitudes favoring patient-centeredness’.

**Table 4:** Results related to the theme “Attitudes favoring patient-centeredness”.

Theme: 1. Attitudes favoring Patient-Centeredness.		
Sub-Themes	Labels	Quotes
a. Seeking the uniqueness of the patient	For shared decision-making, it’s necessary to understand the patient’s point of view	1.a.i-*To put the patient at the center of care, one must first understand their point of view; when faced with a patient who refuses to undergo therapy, one must ask why they do not want to take this medicine? They express their motivation, you try to understand, and then you try to find a new solution, a new way to get them to choose to take the medicine. You explain why their motivation is not entirely right and what the benefit and risk of that medicine are. So you help them make the right choice, but first you have to listen to them.* [INT_ 9]
	Putting the patient at the center means listening to them	1.a.ii-*To put the patient at the center, you must listen to them, without judging them, so they can express themselves freely.* [INT_ 8]
	To ensure patient-centered care, it’s necessary to go beyond the standard	1.a.iii-*To promote patient-centeredness, you need to go beyond the standard, which doesn’t mean not respecting it but not stopping there, applying it to the person recognizing their unique history*. [INT_ 12]
b. Informing the patient and involving them in decision-making	The patient’s knowledge of their own clinical condition is essential to placing them at the center of care	1.b.i- *If I were the patient, I would want to understand what’s happening to prepare myself for the future. I think being informed is fundamental, especially because I am at the center; in my experience, I have met people who were not well informed about the surgical procedure they were undergoing, and putting myself in their shoes, I felt a sense of discomfort, I felt it was not right*. [INT_ 9]
	Informing the patient about the different therapeutic possibilities and letting them choose means putting them at the center	1.b.ii*-In my opinion, the person should be the one to choose what should be done in the care, what we should do is present them with all the possibilities, help them make the best decision for them, but still let them decide or at least involve them in the decision.* [INT_ 9]
	If the patient is informed, they can become a great ally	1.b.iii*-If the patient is educated about the condition, treatments, about everything regarding their health at that moment, I think it’s of great help for professionals, they become a great ally.* [INT_ 3]
	Choices should be discussed with the patient, and the goal of the treatment shared, so that the treatment makes sense	1.b.iv*-Therapeutic choices should be discussed, the end of the care path should be shared with the patient, otherwise, it doesn’t make sense.* [INT_ 3]
	Trust and the absence of hierarchy in the relationship create openness for the patient to ask questions, express doubts, and speak up.	1.b.v*-The use of technical language and an attitude of superiority create distance and don’t leave space for the patient to express their doubts. There is still the stereotype of the hierarchical relationship where the patient feels intimidated and doesn’t ask questions or express doubts.* [INT_ 11]
c. Understanding and managing the patient’s emotions	To put the patient at the center, it’s necessary to understand the emotions they are experiencing	1.c.i-*To promote patient-centeredness, you need to try to understand the emotions they’re feeling, try to put yourself in their shoes to help them.* [INT_ 7]
	To understand the emotional aspects of the patient, it’s necessary to enter into dialogue with them	1.c.ii*-You need to make the patient comfortable if you want to understand their emotions; you need to first establish a trusting relationship, perhaps by focusing on their physical condition first.* [INT_ 2]
	Putting oneself in the patient’s shoes helps understand what the right thing to do is	1.c.iii*-In my experience, I have met a foreign person who didn’t understand Italian, with whom communication was very difficult. How do you know if what you’re doing is the right thing for them? A first step is to try to put yourself in their shoes.* [INT_ 5]
	Openness to emotions in the relationship is an attitude that the professional must exercise with all patients without creating inequalities	1.c.iv*-With some people, managing the emotional side may be easier, but as professionals, we must maintain this attitude of openness with everyone indiscriminately.* [INT_ 7]

From the students’ perspective, to center the patient in care, it is necessary to understand their unique point of view (1.a.i). To do this, the professional must strip away prejudices and adopt an open and listening attitude (1.a.ii). Seeking the uniqueness of the patient means going beyond the standard, not in the sense of eliminating or disrespecting it, but in the sense that in practice, space must also be given to the person’s unique history (1.a.iii); personalizing care is possible without eliminating the standard and improves quality(1.a.iii).

Another attitude that fosters patient-centeredness is involving the patient in the diagnostic and therapeutic process through information and shared decision-making (1.b.i). The patient is capable of making choices, with the decision seen as the outcome of a shared process (1.b.iv). Only through complete information about all possible choices (1.b.ii), using understandable language, can the patient become an ally (1.b.iii) and increase compliance. Additionally, alliance presupposes trust and absence of hierarchies (1.b.v).

Furthermore, students report on the importance of the emotional aspect. Professionals center the patient by empathizing and trying to understand their emotions (1.c.i; 1.c.iv). Engaging in dialogue with the patient and understanding their reactions, emotions, and fears (1.c.ii) allows the professional to guide the person in making the right choice for them (1.c.iii).

#### The emotional bond in the care relationship.

The theme ‘The emotional bond in the care relationship’ includes sub-themes such as the attributes of the emotional bond, the dedication of the professional, and the management of personal emotions; these sub-themes, labels and quotes are shown in Table 5.

**Table 5:** Results related to the theme “The emotional bond in the care relationship”.

Theme 2. The emotional bond in the care relationship		
Sub-themes	Labels	Quotes
a. The attributes of the emotional bond	Creating an emotional bond with the patient creates well-being for the patient and facilitates acceptance of the illness and the care path.	2.a.i*-An emotional bond with the patient facilitates our intervention, and communication becomes simpler as well because if they trust us, thus establishing a relationship of trust, they feel more relaxed, and we lower defenses, reducing distances.* [INT_5]
	Openness to emotions is not a standard but must be managed based on the person in front of me.	2.a.ii*-As you were saying, it’s important not to standardize; each person is unique... Understanding what the person is experiencing at that moment is a step forward... A phrase that struck me a lot from clinical psychology lessons is that we must ‘touch without being overwhelmed’... I think this phrase is very true... and it always remains... that line that acts as a barrier to not be overwhelmed... but still allows you to touch the patient’s emotion.* [INT_12]
	Emotional involvement in the relationship must remain within the boundaries of being a professional.	2.a.iii*-I think there is a certain interpersonal distance that must be maintained because we are professionals; the distance should not be exaggerated, just enough not to bring home what is negative. I think finding that right line that marks the boundary between me and the other person is still part of our profession.* [INT_11]
	The emotional bond between patient and professional is bidirectional.	2.a.iv*-Understanding emotions... it shouldn’t be one-sided... I think there is an exchange between patient and operator... in fact, there must be an exchange... it shouldn’t be one-way... even from the patient, a certain openness and understanding are expected.* [INT_6]
b. The dedication of health professional	Learning to manage emotions in the caregiving relationship is a training, it requires commitment and effort.	2.b.i*-Creating an emotional bond requires a lot of commitment and training... Some find it hard to bond, while others struggle to detach... It takes a lot of effort and willpower. Patients are unique, but so are we; not all professionals are alike in this regard.* [INT_4]
	Creating an emotional bond is a double-edged sword because it can degenerate into excessive emotional involvement harmful to the professional.	2.b.ii*-Secondly, it’s a double-edged sword because if I become very attached, I bring all their emotions home... I get too involved in the situation; if you get too attached, you also risk being influenced by certain things and not remaining objective in front of them. I try to take it in as if I were a friend, but it’s difficult for me to stay detached from people. It’s about finding the right balance.* [INT_3]
	Being ready to embrace all emotions, maintaining calm and kindness are caring attitudes that require effort and commitment for the professional.	2.b.iii*-Sometimes the emotions of the patient or family member are anger and refusal. I think it’s important to welcome them while maintaining calm and a gentle attitude in these cases.* [INT_9]
	Excessive emotional involvement can negatively influence clinical practice.	2.b.iv*-It’s a very fine line because if you go beyond it, there’s a danger of no longer seeing the situation with a clinical eye, and perhaps some details are overlooked. They are a patient, but they shouldn’t become a friend.* [INT_4]
c. The management of the professional’s emotions	Knowing how to manage one’s emotions facilitates empathy and the well-being of professionals.	2.c.i*-Regarding our emotions? You need to know how to maintain a balance... that is, to put yourself in the patient’s shoes, to think as if I were the patient, but without carrying his emotions inside... we are human beings and we have our emotions, but we must try to control them in front of the patients.* [INT_7]
	Knowing how to manage one’s emotions is important to be a point of reference for the patient and the family.	2.c.ii*-It’s important to know how to manage our emotions to be able to help others... to be the anchor for family members, I have to be strong, I can’t think of crying with them even though that moment may not be good for me... excess from either side is not good.* [INT_7]
	In the relationship with the patient, it is important that the professional can also express their own emotions without letting them become an obstacle.	2.c.iii*-It’s right that the emotions we feel in front of patients are expressed... that is, we communicate emotional connection... in this way, the patient feels more confident and calm... of course, all this without being overwhelmed, otherwise, we wouldn’t be rational anymore and we wouldn’t act for the patient’s good... but showing him that we take care of him even from an emotional point of view, in my opinion, is important.* [INT_11]
	The professional also needs to recognize their own humanity and be aware of their emotions to maintain a beneficial relationship with the patient.	2.c.iv*-It’s important to be aware of what we are feeling towards patients, even when they are challenging, especially when we struggle to relate; only with awareness of our emotions can we make that relationship beneficial.* [INT_1]

According to the students, creating an emotional bond with the patient promotes well-being for the patient and facilitates acceptance of the illness and the treatment process (2.a.i); it is an attitude that should be ensured for everyone but tailored according to the individual (2.a.ii). The exchange of emotions is bidirectional (2.a.iv), but while there may be no boundaries for the patient, as they can invest fully in the relationship, for the professional, there is the limit of their role that must be respected (2.a.iii). In this sense, the emotional bond becomes a double-edged sword: if those boundaries are not respected, it can degenerate into excessive emotional involvement harmful to the professional and negatively influence clinical practice (2.b.ii). Being ready to embrace all emotions, maintaining calm and kindness are empathetic attitudes that are learned and requires training and effort (2.b.i; 2b.iii), especially in the face of the patient’s anger and refusal, as well as that of the family members (2.b.ii); humor sometimes represents a strategy of comfort (2.b.v).

Closely linked to the emotional bond with the patient is the management of one’s own emotions. It is important for the professional to be aware of their own emotions so that they can establish a beneficial bond with the patient (2.c.iv). The professional can express emotions in the relationship with the patient but while respecting the limit of professionalism (2.c.iii). The ability to manage one’s own emotions facilitates empathy and promotes the well-being of the professional (2.c.i) and is also beneficial for the patient and their family members, who in a moment of emotional fragility may see the professional as a reference point (23.c.ii).

#### Learning from the patient’s history.

The theme and its components are represented in Table 6.

**Table 6:** Results related to the theme “Learning from the patient’s history”.

Theme 3. Learning from the patient’s history.	
Sub-theme	Quotes
Meetings with patient as teacher help to reflect on one’s own clinical practice and correct errors.	3.i*-In my opinion, meetings with patient and caregiver trainers are very useful; they become a tool for analyzing what you can do well in the next internship, what you can do well at work, what you’ve done wrong. Also, because in internships, you never received feedback from the patient; they leave, and no one knows what they think or what they thought. Instead, with this experience, you can reflect a lot on yourself, understand where others have gone wrong, and avoid making the same mistakes... understanding the critical points, the attitudes, somewhat ineffective ways of taking care.* [INT_12]
Meetings with patient as teacher have helped to gain awareness that the care journey is strongly influenced by their history and their way of reacting.	3.ii*-In these meetings, I acquired awareness of how what can happen to a person during illness and treatment can influence their life, and how their history can influence what they are experiencing, how they approach what awaits them. In my opinion, the important part of taking care of the person is always considering their history.* [INT_12]
Through group work and dialogue with the patient, we arrived at a common line of thought.	3.iii*-During the group work, the caregiver trainer was with us; we answered the questions that the tutors had prepared. Each gave their answer, and with the caregiver, we finally reached a common line... we were all on the same page.* [INT_8]
The history of the patient as teacher is rich in emotional knowledge.	3.iv*-The patient becomes the expert of what they have; the combination of scientific and emotional knowledge is beautiful. It was the turning point of the experience with the patient trainer; no one better than him can explain how one might feel.* [INT_8]

Reflecting on the workshop, the students stated that it helped them in meeting with the patient and caregiver trainer, helping them to gain awareness that the treatment process is strongly influenced by their history and way of reacting (3.ii), and to identify and correct the errors seen in the daily practice of ineffective patient management (3.i). ‘No one better than the patient can explain how one might feel,’ says one student (3.iv); engaging in dialogue with the patient trainer leads them towards a shared understanding, a principle of partnership (3.iii).

### Integration of the quantitative and qualitative finding

The Joint display that integrates the qualitative and quantitative findings is reported in Table 7. There was agreement between quantitative and qualitative data.

**Table 7:** Joint display for qualitative and quantitative data integration.

Outcomes	Quantitative findings	Qualitative findings using labels	Agreement/ Disagreement	Interpretation of meanings across the findings
Patient-centredness	Subscale ‘Sharing’ m pre- = 3.86 m post- = 4.17 t(df)=−4.45 (135) p < 0.001*	Putting the patient at the center means listening to them	Agreement	To prioritize the patient in healthcare means adopting a participatory approach to decision-making in the care pathway and sharing clinical information. The healthcare professional should understand and respect the patient’s viewpoint; the patient should be informed about all therapeutic possibilities and guided in choosing what is best for them. This creates a relationship of trust and alliance.
		The patient’s knowledge of their own clinical condition is essential to placing them at the center of care		
		Informing the patient about what is being done increases trust and compliance		
		If the patient is informed, they can become a great ally		
	Subscale ‘Caring’ m pre- = 4.90 m post- = 5.08 t(df)=−3.35 (136) p < 0.001*	For shared decision-making, it’s necessary to understand the patient’s point of view	Agreement	The centrality of the patient can only be achieved if the healthcare professional goes beyond technicalities and standards, seeking the uniqueness of the patient and their history. In addition to considering these aspects in care, it is important to understand the emotions they experience and pay attention to their reactions and behaviors. Calmness and kindness are attitudes that promote this, as well as trust and the absence of hierarchy, which foster a caring relationship in this sense.
		To put the patient at the center, it’s necessary to understand the emotions they are experiencing		
		To understand the emotional aspects of the patient, it’s necessary to enter into dialogue with them		
		Putting oneself in the patient’s shoes helps understand what the right thing to do is		
		Trust and the absence of hierarchy in the relationship create openness for the patient to ask questions, express doubts, and speak up		
		Being ready to embrace all emotions, maintaining calm and kindness are caring attitudes that require effort and commitment for the professional.		
Empathy	JSEHPS m pre- = 111.94 m post- = 118.34 z = −7.62 p < 0.001*	Creating an emotional bond with the patient creates well-being for the patient and facilitates acceptance of the illness and the care path.	Agreement	Understanding the emotional state of patients and their families by healthcare professionals is part of care and facilitates a patient-centered practice. It’s an attitude that professionals should have with all those they assist, but personalized to the relationship. Empathy is learned and requires effort and commitment; moreover, it must be exercised within professional boundaries, as excessive emotional involvement can harm both the professional and the patient. The attributes of the emotional bond with the patient include uniqueness, bidirectionality, and professionalism. It requires dedication from the professional and is not without risks.
		Openness to emotions is not a standard but must be managed based on the person in front of me.		
		Emotional involvement in the relationship must remain within the boundaries of being a professional.		
		The emotional bond between patient and professional is bidirectional.		
		Learning to manage emotions in the caregiving relationship is a training, it requires commitment and effort.		
		Excessive emotional involvement can negatively influence clinical practice.		
		Humor helps build a relationship of trust but not always and with everyone.		
Emotional Intelligence	SREIT m pre- = 111.51 m post- = 114.70 t(df)=−5.10 (137) p < 0.001*	Knowing how to manage one’s emotions facilitates empathy and the well-being of professionals.	Agreement	The emotional bond with the patient includes the emotions of the healthcare professional. The professional may express their own emotions in the caregiving relationship but must be capable of managing them to maintain a beneficial rapport with the patient. Emotional intelligence involves the perception, evaluation, and expression of emotions, as well as the ability to access and/or generate emotions that can facilitate the relationship with the patient and their family. Being able to manage one’s own emotions facilitates openness to the emotions of others and, therefore, empathy.
		In the relationship with the patient, it is important that the professional can also express their own emotions without letting them become an obstacle.		
		The professional also needs to recognize their own humanity and be aware of their emotions to maintain a beneficial relationship with the patient.		
		Creating an emotional bond is a double-edged sword because it can degenerate into excessive emotional involvement harmful to the professional.		
		Knowing how to manage one’s emotions is important to be a point of reference for the patient and the family.		

### Comparison across academic years (third research question)

#### Description of students of nursing degree program.

To assess the progression of outcomes across academic years, 149 first-year students and 129 second-year students were recruited, in addition to the 146 third-year students, resulting in a total sample of 424 students. The response rate was 98.65% for the first year (number of eligible students = 149) and 100% for the second year.

In all academic years, there is a prevalence of female students (80.42%), and almost all of the sample is of Italian nationality (91.67%). The mean age is 22.67 years (SD 5.86) with a range from 18 to 59 years, and it increases by approximately one year across academic years.

#### Results of the PPOS-8-IT, JESHPS, and SREIT Scales.

The scores of the PPOS-8-IT, JESHPS, and SREIT scales of third-year students post-intervention were compared with the scores of first and second-year students. As the data did not follow a normal distribution for any of the three scales (Shapiro-Wilks test p < 0.001; Kolmogorov test p < 0.05), the non-parametric Kruskal-Wallis test was used for comparing the three academic years (Table 8).

**Table 8:** Comparison of outcomes across three academic years.

Scales			Academic Year			H (df)	p-value
		I	II	III (pre-)	III (post-)		
**PPOS-8-IT Total score**	**M**	4.00	4.25	4.37	4.63	55.99 (2)	<0.001
	**IQR**	4.50	3.00	3.00	3.00		
	**m**	4.03	4.26	4.38	4.62		
	**DS**	0.64	0.59	0.60	0.64		
**JSEHPS Total score**	**M**	113.00	114.00	114.00	120.50	37.78 (2)	<0.001
	**IQR**	96.00	65.00	15.00	13.75		
	**m**	111.85	113.02	111.94	118.34		
	**DS**	11.71	9.58	11.47	12.58		
**SREIT Total score**	**M**	112.00	114.00	112.00	116.00	4.83 (2)	0.09
	**IQR**	73.00	66.00	20.00	79.00		
	**m**	112.02	113.98	111.51	115.16		
	**DS**	13.14	11.41	14.12	14.15		

Legend: M = Median; IQR = Interquartile Range; m = Mean; DS = Standard Deviation; H = Kruskal-Wallis test; gl = Degrees of Freedom; *p < 0.05..

Results show an increase in scores with increasing academic years for all three scales. The analyses reveal that the difference among the three years is statistically significant both for the PPOS-8_IT scale (H(2)=55.99, p < 0.001) and the JSEHPS scale (H(2)=37.78, p < 0.001). For the SREIT scale, scores increase with academic year, but not significantly (H(2)=4.83, p < 0.09). However, when comparing the scores of the first and second years with the scores of the third year pre-intervention, although the scores are higher, statistical significance is only reached for the PPOS-8_IT scale (H(2)=23.20, p < 0.001), but not for the JESHPS (H(2)=0.362, p < 0.865). For the SREIT scale, the mean score of the third year pre-intervention (m = 111.51 ± 14.12) is lower than the mean score of the first year (m = 112.02 ± 13.14) and the second year (m = 113.98 ± 11.41).

## Discussion

The primary aim of this study was to assess the impact of an educational intervention involving a patient and caregiver as teachers on patient-centered attitudes, empathy, and emotional intelligence in nursing students. The results show significant improvements in all three outcomes, supporting the effectiveness of the intervention.When compared with a previous study conducted at the same university—albeit involving different students and a different workshop—our findings remain consistent regarding both patient-centeredness [46] and empathy [23]..

Patient-centeredness, is reported as the main outcome in numerous international studies [47–50].

As Scholl et al. (2014) highlight [3], this concept involves recognizing the patient as a person, developing self-awareness, overcoming biases and stereotypes, embracing multiple perspectives, bridging theory and practice, and cultivating a humanistic approach. Our results support these components, with qualitative data underscoring the transformative impact of involving patients and caregivers in the educational process.

One key aspect of patient-centeredness is the involvement of patients in decision-making through information-sharing and participatory dialogue. Our findings reflect this through the ‘Sharing’ subscale of the PPOS-8-IT and are reinforced by qualitative data suggesting that true patient-centered care entails open communication, mutual trust, and shared decision-making. Likewise, the ‘Caring’ dimension emphasizes sensitivity to the patient’s emotions, expectations, and personal circumstances, which is essential in establishing a meaningful therapeutic relationship [4]. Patient and caregiver participation in education plays a pivotal role in cultivating this sensitivity [48].

Integrated findings from our study suggest that achieving patient-centeredness requires moving beyond technical skills and standard procedures, embracing the uniqueness of each patient’s story. This includes understanding patient emotions and responding with calmness, kindness, and humility, all of which contribute to a respectful and supportive care environment.

Our results also show that involving patients and caregivers is effective in promoting empathy in nursing students. These findings are in line with both national [23] and international research, such as Heidke et al. (2018) [51], who found that recorded interviews with healthcare consumers significantly improved empathy among first-year nursing students. Consistent with literature on experiential learning as a tool to enhance empathy [7], our students displayed moderate baseline levels that significantly improved post-intervention, confirming evidence from similar studies [52,53].What our study adds—through the integration of qualitative and quantitative data—is a deeper understanding of how recognizing patients’ and families’ emotional states contributes to care and supports a patient-centered approach. Empathy, while learnable, requires conscious effort, consistent practice, and clear professional boundaries to avoid emotional overinvolvement. The emotional bond between patient and professional is marked by uniqueness, mutual engagement, and professionalism, demanding dedication and emotional awareness.

While literature supports the value of emotional competence in nursing, the use of patients as co-teachers remains underexplored and not widely recognized as an effective pedagogical strategy to promote emotional skills [14]. Our study contributes to filling this gap by demonstrating its potential impact. Emotional self-management is another key component, closely tied to emotional intelligence. Prior research has linked emotional intelligence with effective emotional regulation in healthcare settings [28,30]. This study is the first to examine the impact of involving patients and caregivers in nursing education on students’ emotional intelligence. Our findings show a significant increase in emotional intelligence post-intervention, though not across all items. While a single educational activity may not fully develop emotional intelligence, our integrated results suggest that the emotional bond between professionals and patients includes the professionals’ own emotions. These must be acknowledged and managed to sustain a healthy and effective care relationship.

Emotional intelligence enhances openness to others’ emotions, supporting the development of empathy. Our findings align with literature indicating that emotionally intelligent students establish stronger relationships with patients and families and are better at managing emotional challenges [28,54,55]. Emotional intelligence also supports self-compassion, making students more attentive to emotional cues, which in turn improves communication and patient interaction [56].

As for learning mechanisms, our findings suggest that patient and caregiver involvement in education prompts students to recognize how patients’ histories and emotional experiences influence the care journey. This reflection encourages self-awareness and the correction of clinical practice errors. As documented in the literature, storytelling represents an interpersonal exchange fostering empathy and transformative learning [57–59]. In our previous study, this process was defined as a dialogue between patient and professional that helps dismantle stereotypes and enrich understanding [58].

An interesting outcome from this study is the observed improvement in patient-centered attitudes, empathy, and emotional intelligence across the three years of the program. This contrasts with previous studies reporting a decline in these traits, often attributed to an overly biomedical educational culture [20,24]. Our results suggest that the educational intervention may counteract this trend, especially regarding empathy and patient-centeredness. Regarding emotional intelligence, our findings are consistent with literature suggesting a natural developmental progression throughout the course [60].

This study presents several strengths. First, the involvement of patients and caregivers as co-teachers offers an innovative and person-centered educational approach that is still underexplored in nursing education. The use of a mixed-methods design allowed for both the objective measurement of outcomes and the exploration of students’ subjective experiences, providing a comprehensive understanding of the intervention’s impact. The integration of validated instruments and a rigorous analytic framework—combining statistical tests and thematic analysis—adds credibility to the findings. Moreover, the triangulation of quantitative and qualitative data reinforces the validity and depth of interpretation. Finally, the results offer meaningful insights for educators seeking to design curricula that foster emotional competencies and patient-centered attitudes across all academic years.

## Supporting information

S1 File Syllabus of the educational intervention. (PDF)

S2 File Focus Group Guide. (PDF)

S3 File Quantitative Data Set. (XLSX)
