# Supplementary material for: Enhancing nursing students’ patient-centeredness attitudes and emotional skills through co-teaching with patients and caregivers: A mixed-methods study
Source: PLoS One. 2025 Sep 29;20(9):e0332510. doi: 10.1371/journal.pone.0332510 (PMC12478891; doi:10.1371/journal.pone.0332510)
Supplement: S1 File — (PDF) [file pone.0332510.s001.pdf]

S1 File. Syllabus of the educational intervention

WORKSHOP ‘ComuniCare: Partnership between Patient and Caregiver’

3rd Year – Nursing Degree Program
Academic Year 2023-2024
University of Modena and Reggio Emilia - Modena Campus

Learning Objectives:

- The student will be able to listen to and understand the stories of patients and caregivers, recognizing thoughts, feelings, key points, and relational elements of care.
- The student will value the history, expectations, emotions, and life circumstances of the patient and caregiver as essential elements in the care relationship, identifying attitudes, behaviors, and communicative elements (verbal and non-verbal) that facilitate or hinder caring.
- The student, during and at the end of the lesson, will be able to recognize the understanding of the emotional state of the patient and caregiver as an important component in the care relationship that influences the health status of the patient/caregiver and the outcomes of care. They will recognize attitudes, behaviors, and communicative elements (verbal and non-verbal) that facilitate or hinder the empathic relationship.
- The student, during and at the end of the lesson, will be able to recognize the patient and caregiver as a resource in the decision-making process of care, identifying attitudes, behaviors, and communicative elements (verbal and non-verbal) that facilitate or hinder the involvement of the patient and caregiver in the care process.
- The student will gain greater awareness of how to recognize, use, understand, and consciously manage their own and others' emotions.

Teaching Methodology:

A workshop co-led by professional teaching tutors, a patient, and a trained caregiver, which includes:

- Workshop Opening (9:00-9:20)
- Storytelling or Narrative Interview (9:20-10:00)
- Group Work (10:00-10:50) (4 groups of about 18 students each)
- Break (10:50-11:00)

- Group Work Presentation and Conclusions (8 minutes per group, 11:00-12:00)
- Post-Workshop Data Collection (12:00-13:00)

Dates and Times:

- 1st Group: Thursday, October 5, 2023, from 9:00 to 13:00
- 2nd Group: Friday, October 6, 2023, from 9:00 to 13:00
