# Supplementary material for: Enhancing nursing students’ patient-centeredness attitudes and emotional skills through co-teaching with patients and caregivers: A mixed-methods study
Source: PLoS One. 2025 Sep 29;20(9):e0332510. doi: 10.1371/journal.pone.0332510 (PMC12478891; doi:10.1371/journal.pone.0332510)
Supplement: S2 File — (PDF) [file pone.0332510.s002.pdf]

S2 File. Focus Group Guide

1. Opening

- Introduction of the lead researcher and moderator.
- Explanation of the research purpose.
- Setting ground rules for the session and requesting consent for recording.
- Clarification of the specific objectives of the meeting: ‘what is expected from this focus group.’

2. Discussion

- What does it mean to you to place the patient and/or caregiver at the center of care?
- How can a healthcare professional act in the relationship with the patient to place them at the center of care?
- What can hinder and/or facilitate patient-centered care?
- What role do the emotions of the professional play in the relationship with the patient and/or caregiver, in your opinion? What role do the emotions of the patient and caregiver play?
- What struck you the most about today's experience?
- How do you think this lesson was useful? What did it serve?
- How was the interaction with the patient/caregiver trainers?
- How do you think this experience might modify or has modified your professional practice?

3. Closing phase

- Ask participants if there are any additional observations or comments they would like to express.
- Summarize the main points that emerged from the discussion.
- Thank the participants for their participation, informing them that they might be contacted again during the analysis and discussion phase of the results.
